# Variation in Pea (*Pisum sativum* L.) Seed Quality Traits Defined by Physicochemical Functional Properties

**DOI:** 10.3390/foods8110570

**Published:** 2019-11-13

**Authors:** Carla S. Santos, Bruna Carbas, Ana Castanho, Marta W. Vasconcelos, Maria Carlota Vaz Patto, Claire Domoney, Carla Brites

**Affiliations:** 1 Universidade Católica Portuguesa, CBQF—Centro de Biotecnologia e Química Fina—Laboratório Associado, Escola Superior de Biotecnologia, Rua Diogo Botelho 1327, 4169-005 Porto, Portugalmvasconcelos@porto.ucp.pt (M.W.V.); 2 INIAV—Instituto Nacional de Investigação Agrária e Veterinária, Avenida da República, 2780-157 Oeiras, Portugal; bruna.carbas@iniav.pt (B.C.); anavargascastanho@gmail.com (A.C.); 3 ITQB NOVA—Instituto de Tecnologia Química e Biológica António Xavier, Universidade Nova de Lisboa, Avenida da República, 2780-157 Oeiras, Portugal; cpatto@itqb.unl.pt; 4 John Innes Centre, Norwich Research Park, Norwich NR4 7UH, UK; claire.domoney@jic.ac.uk

**Keywords:** cooking quality, genetic markers, pea flour, protein, pulses, rapid visco analyser profile, resistant starch, seed phenotype

## Abstract

Pea is one of the most produced and consumed pulse crops around the world. The study of genetic variability within pea germplasm is an important tool to identify outstanding accessions with optimal functional and nutritional qualities. In the present study, a collection of 105 pea accessions was analysed for physicochemical properties, pasting viscosity, and basic composition parameters. While pasting viscosities were negatively correlated to hydration capacity, cooking time, and basic composition, a positive correlation was found between the hydration capacity and the basic composition parameters. Basic composition (protein, fibre, fat, and resistant starch) parameters were further evaluated regarding seed trait morphology, namely, seed shape, colour, and surface. Allelic characterisation at the *r* and *rb* genetic loci was performed in a subgroup of 32 accessions (3 phenotyped as smooth and 29 as rough seeded), revealing that none of the initially classified rough-seeded accessions were *rb* mutants, 19 were *r* mutants, and 13 were neither *r* nor *rb*. Despite their initial phenotypic classification, the 13 accessions genetically classified as smooth behaved differently (*p* < 0.05) to the 19 *r* mutants in terms of physicochemical properties, pasting viscosity, and basic composition parameters. Using multivariate analysis of the most discriminatory parameters for the food-related traits studied, the best-performing accessions at functional and nutritional levels were identified for future plant breeding to improve field pea production and consumption.

## 1. Introduction

Field pea (*Pisum sativum*) is an increasingly important legume crop grown around the world, having a total harvested area of 8 million ha and a total production of 16 million tonnes per year [1]. Currently, Europe secures a pea production share of about 44%, followed by America and Asia. Of the total 5.1 million tonnes of pulses produced in Europe, field pea accounts for more than 40% of the production volume [1,2]. To increase their consumption and avail consumers of their functional and nutritional advantages, legume (pulse) seeds are being processed into flours to be used as ingredients in the preparation of new food formulations [3,4,5]. The seeds of pulse crops have a valuable nutrition profile, with high protein content, minerals, carbohydrates, and fibre [6]. Amongst the carbohydrate fractions, the most abundant component is starch which, in the case of pulses, has a low glycemic index [7]. The low digestibility of starch from pulses is one of the main reasons why they can provide high nutritional value to humans, given that part of the starch fraction—resistant starch—is not digested by the small intestine and functions similarly to dietary fibres, accounting for its several health benefits [8]. Additionally, the lack of gluten proteins in pulse seeds is very important in helping to meet the demands of gluten-free diets for people who suffer from celiac disease [8].

One important characteristic that influences the acceptability of legume food products relates to food texture and sensory qualities, for which the pasting profile of starch has been shown to be important in different legume species [9]. However, limited studies have focused on variability in the pasting profiles of starch in seeds from large collections of pea germplasm. The pasting properties of food result from the transformation of starch granules through the application of heat in the presence of water. Since certain seed quality characteristics like the cooking behaviour or the proximate composition of different pea accessions can vary according to seed traits like size, shape, or colour [10,11], it is important to understand the relationships between these factors and such characteristics [12]. The profile of pea starch pasting varies significantly among pea cultivars [12,13]. Both genotype and environment were found to exert a significant impact on the flour pasting characteristics of nine field pea genotypes harvested from two different growing sites [14,15].

Throughout the continuous selection and breeding of pea, different varieties have been developed for different food and feed uses. Several classes of pea have been bred for food use, the major classes being the vining (seeds harvested as immature vegetable) and combining (harvested as mature dry seed) types. However, the assessment of genetic diversity is crucial for identifying the best accessions for specific end-use requirements [16].

The wrinkled-seeded phenotype in pea has been associated with natural or induced mutations impacting on the concentrations of starch and sucrose; analysis of allelic variation at the *r* and *rb* loci has been used for screening variation in natural germplasm variants [17]. The phenotype of starch grains is also affected by mutations in pea, and compound starch grains are a feature of the natural and induced mutations at the *r* locus which lead to a wrinkled-seeded phenotype [17].

Understanding trait and molecular diversity within pea germplasm will support investigations of the factors which affect the nutritional and functional properties of seeds and is thus of utmost importance to broadening the genetic basis of cultivated peas [15]. The aim of this study was to characterise the variability which exists among pea accessions from a worldwide representative collection of the germplasm used by European breeders, with a focus on their basic composition, resistant starch, pasting, and cooking behaviour, and to correlate these properties with seed morphological (shape, surface, colour) traits. A subset of samples was selected according to seed surface traits and analysed for allelic variation at the *r* and *rb* loci. Overall, the goal is to enable marker-assisted selection for cooking- and eating-quality-associated traits in pea.

## 2. Materials and Methods

### 2.1. Plant Material

For this study, 105 pea (*P. sativum*, PS201-305) accessions were selected from the breeding collection at the IAS-CSIC (Córdoba, Spain) germplasm bank (Table S1). The accessions were multiplied in Córdoba during 2014 under the same field conditions and were irrigated and hand-weeded as needed. Harvest was performed by hand, and seeds were stored at 5 °C until analysis. 

### 2.2. Physicochemical Characteristics and Cooking Time

Water hydration capacity (% HC) and percentages of unhydrated seeds (US) were determined by the AACC 56-35.01 method [18]. Pea sample cooking times (minutes) were estimated by the Mattson Cooker method, as described [9].

### 2.3. Pasting Properties, Resistant Starch, and Basic Composition

Pea seeds were milled (Cyclone Falling 3100 with 0.8 mm mesh; Perten, Hägersten, Sweden) to produce flour.

Pasting properties were determined using a Rapid Visco Analyser according to AACC method 76-21 [19]. Pasting analysis was conducted on duplicate flour samples (3 g in 25 mL water) held at 50 °C for 1 min, heated at 12 °C/min to 95 °C, held at 95 °C for 2.5 min, cooled subsequently at 12 °C/min to 50 °C, and held at 50 °C for 3 min. Peak, trough, breakdown, final viscosity, and setback from trough were expressed in centipoise (cP).

The resistant starch was analysed according to AACC method 32–40.01 [20] using an assay kit (Megazyme International, Bray, Ireland). 

Contents of protein, fibre, and fat were assessed using a near-infrared (NIR) analyser (MPA; Bruker, Billerica, MA, USA), with ground flour calibrations for grain legumes provided by Bruker (*n* > 500; *R*^2^ > 90). The NIR data were validated with 10% of the samples selected to cover the range and characterised by the reference methods: protein by the combustion method ISO 16634-2:2016 [21], calculated by multiplying the nitrogen concentration by a conversion factor of 6.25; fat extracted by using petroleum ether in a Soxhlet apparatus according to ISO 6492:1999 [22]; and fibre by the intermediate filtration method, ISO 6865:2000 [23].

### 2.4. Seed Trait Classification

**Figure 1:**
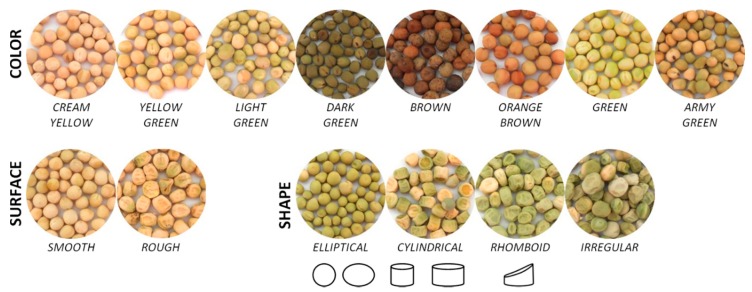
Pea seed categories defined for colour, surface, and shape. Geometric representations correspond to seed shape (elliptical, cylindrical and rhomboid).

Pea seeds were classified according to different morphological traits (Figure 1): shape (elliptical, cylindrical, rhomboid, or irregular), surface (rough or smooth), and colour (cream yellow, yellow green, light green, dark green, brown, orange brown, green, or army green).

A subset of lines (32) selected as having “rough” or “wrinkled” seeds (29 accessions) and three smooth (round-seeded) accessions (259, 286, 289) with low viscosity profiles were genotyped at the *r* and *rb* genetic loci to determine the nature of the mutation which was impacting on the seed surface trait. The *r* locus encodes starch-branching enzyme I, whereas the *rb* locus encodes the large subunit of ADP-glucose pyrophosphrylase [17]. Seed meals were used for the extraction of DNA for 29 samples, and leaf DNA was extracted for the remaining three lines where high-quality DNA could not be extracted. A triplex assay was used to determine the nature of the *r* locus, whereas the *rb* allele was determined by sequencing, using PCR assays as previously described [17]. Starch granule shape appearance was determined by microscopy for this set of lines to validate the genotyping results. Four pea accessions of known genotype were included as controls in the DNA assays (JI 2822, *RRrbrb*, simple starch grains; JI 1194, *rrRbRb*, compound starch grains; JI 281, *RRRbRb*, simple starch grains; JI 399, *RRrbrb*, simple starch grains).

### 2.5. Statistical Analysis

Data were analysed using GraphPad Prism version 8.1.2 for Mac OS X (GraphPad Software, La Jolla California USA, www.graphpad.com). The variation within each seed trait was analysed by one-way analysis of variance (ANOVA). Physicochemical parameters determined for the genetically classified rough- and smooth-seeded accessions were compared by unpaired *t*-tests using the Holm-Šidák method. Statistical significance was considered at *p* < 0.05.

Overall variation of the physicochemical, cooking, rheological, and basic composition characteristics was assessed by principal component analysis (PCA) using Tanagra data mining software, version 1.4.5 (Lyon, France) [24].

## 3. Results and Discussion

### 3.1. Seed and Flour Variation in Pea Germplasm

**Figure 2:**
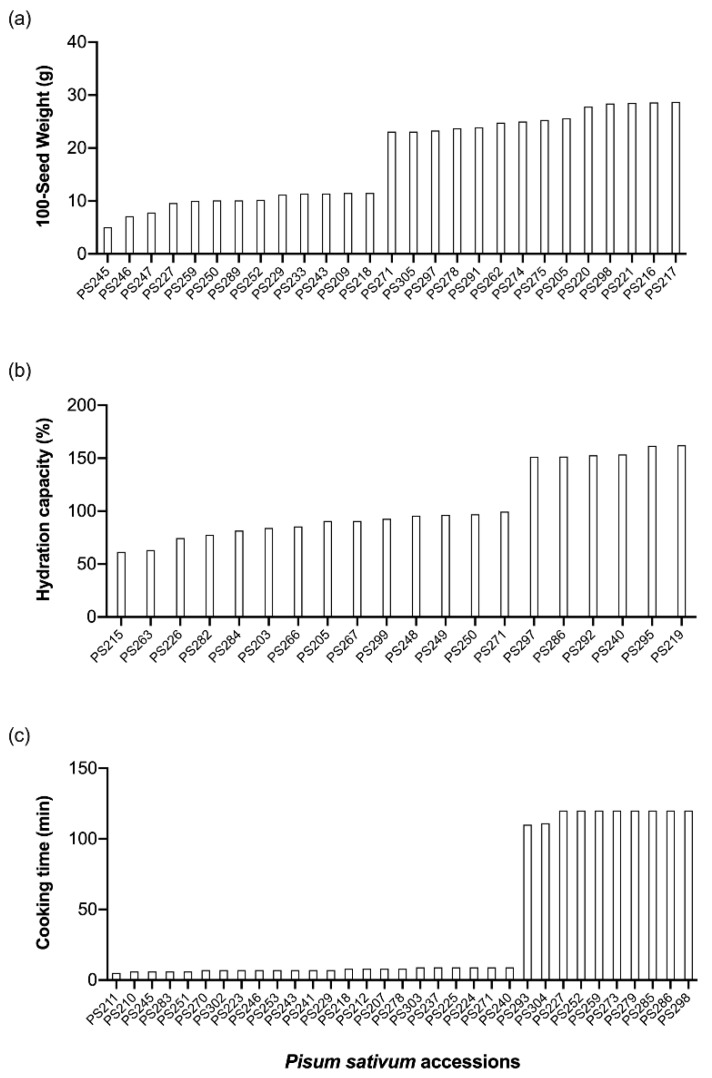
Lowest- and highest-scoring pea (*Pisum sativum*) accessions for (**a**) 100-seed weight: 12 g–23 g; (**b**) hydration capacity: 100%–150%; and (**c**) cooking time: 10 min–100 min.

In the present work, a set of pea accessions (105) was analysed for variation in traits related to food end-use. In terms of 100-seed weight, the accessions 245, 246, 247, and 227 were the ones with lowest weight (below 10 g), while the accessions 298, 221, 216, and 217 registered weights above 28 g (Figure 2a). Low seed weight has been shown to be related to the presence of relatively small-sized starch granules per unit area [25], which might impact hydration capacity and cooking time. The hydration capacity refers to the amount of water absorbed per 100 g of whole mature (dried) seeds. The accessions 246 and 247, which showed the lowest seed weights, also displayed relatively low hydration capacity (Figure 2b) and cooking time (Figure 2c). Despite the variability among the pea collection regarding cooking time (5–120 min), the average of 33 min is low when compared to other pulses [9].

In order to successfully substitute or partially replace wheat flour with pulse flour, it is important to understand how the resulting dough will perform in terms of its rheological properties. Pasting parameters, namely, peak, trough, breakdown, setback, and final viscosities, were analysed as these affect the processing conditions [15]. The analysis of the pasting viscosities (Table 1) showed that the peak viscosity, the maximum viscosity achieved by the samples, ranged between 83 and 4836 cP; the trough viscosity, which represents the decrease in paste viscosity caused by the disruption of starch granules, ranged between 58 and 4066 cP; the breakdown viscosity, the difference between the peak viscosity and trough viscosity, ranged between 8 and 867 cP; the final viscosity ranged from 212 to 7471 cP; and the setback viscosity, the difference between peak and final viscosity, was in the range 151–3489 cP.

The average values of the pasting properties of the 105 pea accessions were similar to those reported by others [14] and to the values obtained for other pulses, such as grass pea, chickpea, or lentil [9]. When compared to wheat varieties [26], the peak viscosity was generally lower, which could be related to differences in the amylose content of starches. In addition, the breakdown viscosity was lower for the pea collection described here (285 cP) when compared to wheat values (669 cP). This might present an advantage when incorporating pea flours into food formulations, since breakdown values are a measure of the degree of paste stability [26], and high breakdown viscosity can reduce the ability of flour to withstand heating during cooking [27]. The lower breakdown viscosities observed here are similar to those recently reported by others for pea and other pulses [28].

On average, the pea accessions contained 22% protein, 7% fibre, and 2% fat—values which are generally comparable to the values reported by others for this pulse [6,12,15,29]. The maximum resistant starch content was 7% in the pea accessions 220, 221, and 228, which also showed higher peak and setback viscosities. Also, the average for the 105 accessions was 3%, which is consistent with the observation that this pulse, when compared to others such as chickpea or lentil, has higher resistant starch percentages [6]. Once again, this is important since higher resistant starch contents lead to slower rates of digestion, enabling the use of pea starches in dietetic foods [30,31].

### 3.2. Correlation Analysis of Pasting, Physicochemical, and Basic Composition Parameters

Correlation coefficients estimated on the means of data from all pea accessions for pasting (trough viscosity, break viscosity, final viscosity, and setback viscosity), physicochemical parameters (100-seed weight, hydration capacity, unhydrated seeds, and cooking time) and basic composition (protein, fibre, fat, and resistant starch contents) are presented in Table 2.

As previously reported [9], all viscosity parameters were positively correlated with each other (*p* < 0.01). These were all positively correlated to 100-seed weight (*p* < 0.05) but showed a significant negative correlation to hydration capacity, as well as cooking time (Table 2). This negative correlation may be due to the fact that viscosity parameters are highly related to the firmness and cooking quality of pulses [32], which is in turn also influenced by the starch composition. Here, the peak, trough, final, and setback viscosities were negatively correlated to resistant starch content (*p* < 0.05).

Further correlations were found when looking at the basic composition. Firstly, all nutritional components were negatively correlated to the viscosity parameters (Table 2). Concordantly, in landraces of *Phaseolus* bean, fat content was shown to be negatively correlated to peak viscosity, but protein content displayed a positive correlation [33]. Additional positive correlations between protein, fibre, fat, resistant starch, and hydration capacity were found (*p* < 0.01), in agreement with the literature [32,34]. Fibre and fat were also positively correlated with cooking time, possibly indicating the major role of these constituents in pea processing.

### 3.3. Seed Trait Variation

The means of the peak, trough, break, final, and setback viscosity, 100-seed weight, hydration capacity, unhydrated seeds, and cooking time for each class of seed shape, surface, and colour are presented in Table 3.

Regarding seed shape, most accessions had ellipsoid (*n* = 42) and cylindrical (*n* = 24) shapes, and these were the types of seeds with higher viscosity values (Table 3). Irregular seeds displayed significantly longer cooking time when compared to ellipsoid or cylindrical seeds (Table 3). Irregular seeds showed higher protein and resistant starch contents when compared to ellipsoid and cylindrical seeds (Table 4).

Regarding seed colour, the variability was not as high for all parameters as it was for seed shape (Table 3). However, most seeds showed a yellow green (*n* = 24) or green (*n* = 23) colour. Significant differences were found between these two groups for peak, trough, final, and setback viscosities, where yellow green seeds had higher values. They also differed in hydration capacity, where yellow green seeds had lower values (Table 3). Analysis of the basic composition variability (Table 4) showed that dark green seeds had higher protein content than cream yellow, and light green seeds had the highest resistant starch content, this difference being significant when compared to brown-coloured seeds.

Variation in seed surface type among the lines was apparent, and the majority of seeds were smooth (*n* = 64). These seeds had higher viscosity parameters (*p* < 0.05) and shorter cooking time (*p* > 0.05) (Table 3). When looking at basic composition, the only significant difference detected between the two seed surface types was that smooth seeds have a lower protein content when compared to rough seeds (Table 4).

### 3.4. Characterisation of Allelic Variation at the r and rb Genetic Loci

For the 32 selected lines, genotyping analysis revealed that none of these were *rb* mutants, 19 were *r* mutants, and 13 were neither *r* nor *rb* (Table 5). The seed granule morphology scores confirmed the genotyping results, where the *r* accessions showed a compound granule structure, and those lines which were neither *r* nor *rb* showed a simple starch granule structure (Table 5). The controls included three rough-seeded lines (JI 1194, JI 2822, and JI 399 as one *r* mutant and two *rb* mutant lines) and one wild-type smooth-seeded line (JI 281).

On the basis of these results, it seems likely that 13 of the 29 classified as having “rough-seeded” phenotypes were genetically round (smooth) seeded and that the three lines classified as having “smooth-seeded” phenotypes (259, 286, 289) with low viscosity profiles were genetically “rough-seeded”.

The 13 lines initially classified as “rough”-seeded which were scored as *RRRbRb*, genetically classified as “smooth”, demonstrated the strong environmental effect on the seed surface trait phenotype which led to difficulties in obtaining consistent classification scores across different growth seasons or generations. It has been shown previously that two mutations (*r*, *rb*) account for the round-/wrinkled-seeded phenotype in a pea germplasm resource [17]. Premature harvest of round-seeded genotypes or premature desiccation as a result of stress will lead to a wrinkled appearance of the seeds when residual water is lost from seeds more rapidly than would be the norm.

**Figure 3:**
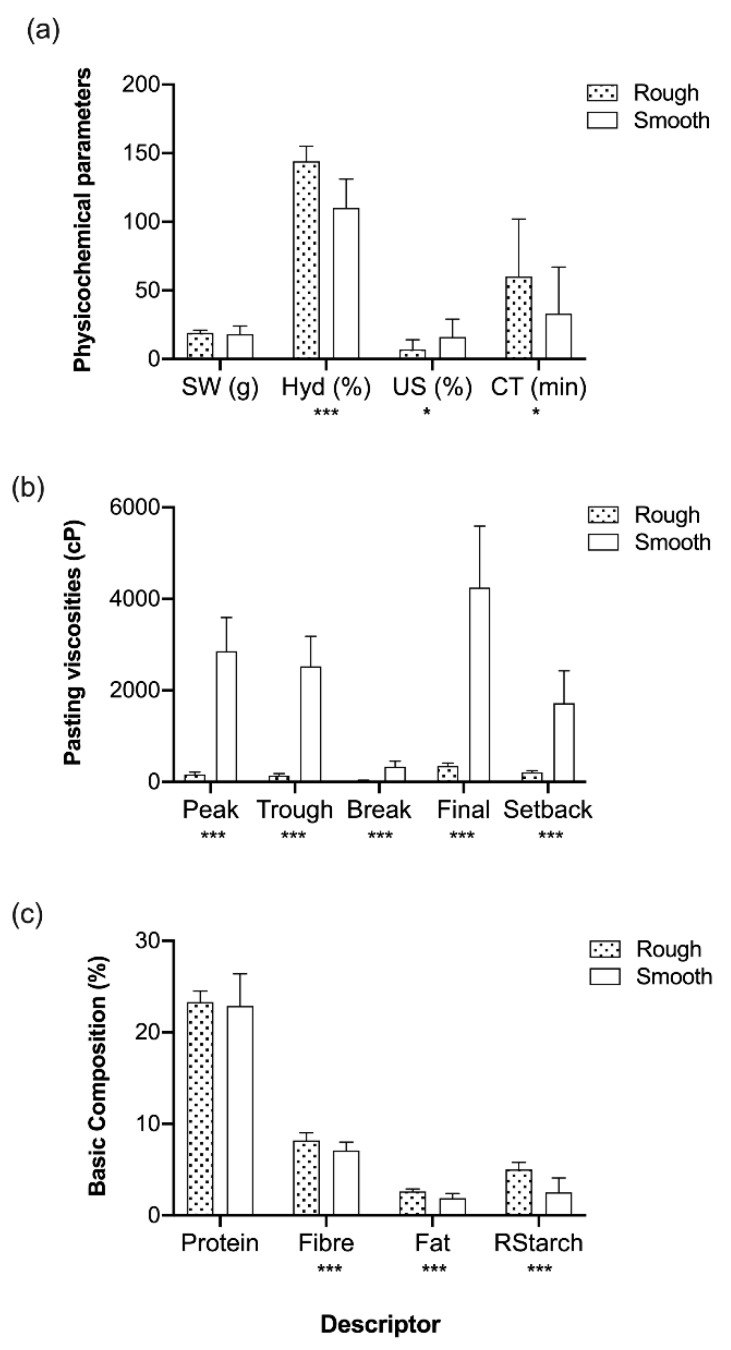
Physicochemical parameters determined for the genetically classified rough- (*n* = 19) and smooth-seeded (*n* = 13) accessions; (**a**) seed weight, SW; hydration capacity, Hyd; unhydrated seeds, US; and cooking time, CT; (**b**) pasting viscosities in centipoise, cP; (**c**) basic composition: protein, fibre, fat, resistant starch (RStarch). Bars are means ± SD. * and *** indicate significant differences at *p* < 0.05 and *p* < 0.001, respectively, by unpaired *t*-tests using the Holm–Šidák method.

These 13 genetically smooth-seeded accessions revealed different behaviour in regards to all of the components analysed in the present study when compared to the 19 rough-seeded accessions (Figure 3). For example, when looking at the physicochemical parameters (Figure 3a), smooth seeds had significantly lower hydration capacity, higher unhydrated seed percentage, and shorter cooking time. Also, in the viscosity profiles (Figure 3b), the smooth-seeded accessions exhibited significantly higher values (with the exception of accession 247). Finally, significant differences were also found between these two groups in basic composition (Figure 3c). Smooth seeds had significantly lower levels of fibre, fat, and resistant starch (but not protein) when compared to the rough-seeded accessions. It is interesting to note that three seed samples (226, 247, 299) posed a problem for DNA preparation, and leaf DNA was required to enable the genotyping assays (Table 5). This may reflect differences, either genetic or environmental, in the nature of starch in the seeds of these lines, which can interfere with the isolation and purification of other seed components. 

The molecular genetic basis for the different viscosity behaviours in the genetically rough-seeded accessions is the *r* mutation and a consequence of the insertion in the starch-branching enzyme I-encoding gene (*sbeI* gene), affecting the carboxy-terminal region of the enzyme and the synthesis of amylopectin in developing pea seeds [35]. The mutation has been widely adopted by the vegetable industry and is the basis for most commercial vining cultivars.

### 3.5. Multivariate Analysis

**Figure 4:**
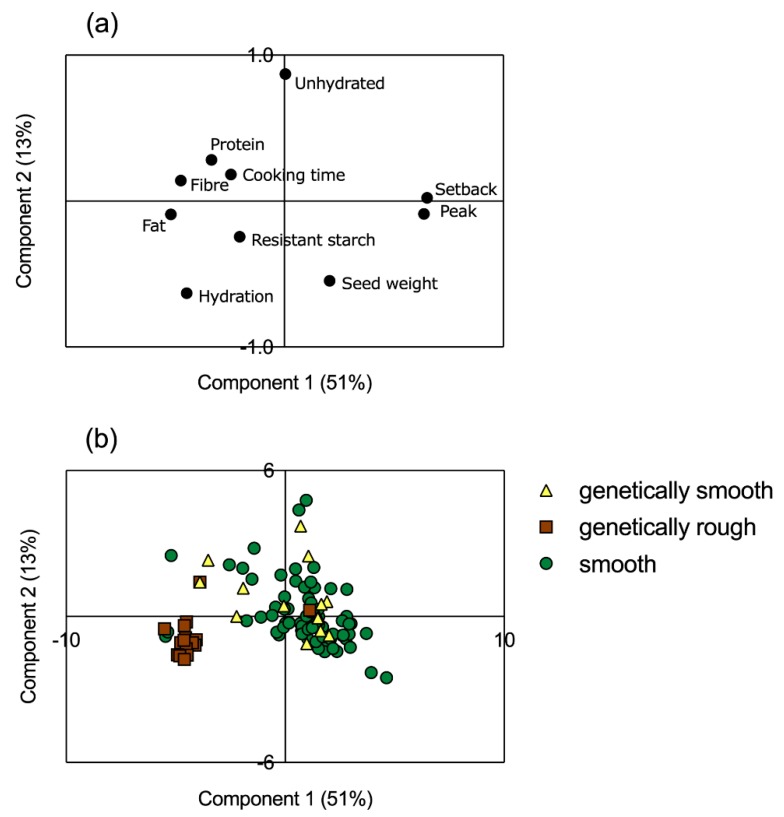
Principal component analysis of the factor loadings (**a**) and of sample scores by accession number (**b**) for the pea samples analysed; yellow triangles represent genetically classified smooth accessions, brown squares represent genetically classified rough accessions, and green circles represent smooth-phenotyped accessions.

Principal component analysis was performed including the peak and setback viscosity values; morphological and cooking parameters, namely, 100-seed weight, hydration capacity, unhydrated seeds, and cooking time; and composition in terms of percentage of protein, fibre, fat, and resistant starch (Figure 4).

The first two components justified 51% (Component 1) and 13% (Component 2) of the total variance, accounting for 64% of the variance. The first component had a positive correlation with basic composition parameters, cooking time, and hydration capacity, while viscosity parameters showed a negative correlation with these parameters (as also observed in Table 2). The second component showed a negative correlation with 100-seed weight and a positive one with the unhydrated seeds parameter (Figure 4a). 

Along the first component, a group of 17 accessions was separated from the rest of the collection (213, 219, 256, 257, 261, 272, 273, 279, 285, 286, 289, 292, 293, 294, 295, 296, 297), and these mostly corresponded to the group of genetically confirmed rough-seeded accessions. Hence, the seed surface trait seems to be highly correlated to fat, resistant starch, and hydration capacity. 

Accessions 220, 221, and 300 showed higher peak and setback viscosity values; accessions 263, 215, and 226 were separated from the group due to their higher percentage of unhydrated seeds; and accession 259 had higher percentages of fibre and fat (Figure 4b).

Moreover, it is possible to confirm that the 13 accessions phenotyped as rough but genetically classified as smooth behave similarly to all 64 accessions initially phenotyped as smooth, and that the 19 accessions confirmed as “rough” constitute a separate group along the first component.

## 4. Conclusions

The present results highlight the value of molecular analyses combined with the study of quality parameters, enabling the selection of appropriate pea germplasm and breeding for discrete end uses.

The pea collection analysed here displayed a favourable pasting profile for the development of flour for baking and other food formulations. Results for protein, fibre, and fat contents were comparable to those from other pulses. Resistant starch values varied greatly, however, among the pea accessions analysed; this component was negatively correlated to the pasting viscosity, an important contributor to cooking property variation.

Phenotype-based characterisation distinguished seeds according to shape, colour, and surface traits. While for the shape and colour classes defined, the results of physicochemical analyses were scattered, in contrast, all parameters differed significantly between the rough- and smooth-seeded classes. Of these, 29 rough- and 3 smooth-seeded accessions were further characterised for their allelic variation at the *r* and *rb* genetic loci. Indeed, 13 of the rough-seeded phenotyped accessions were genetically characterised as smooth, and their physicochemical responses were similar to the behaviour of the other smooth-seeded accessions. 

A final PCA study was performed wherein the pea accessions were separated according to their surface type, linking this trait to cooking and to nutritional value traits, mainly determined by fibre, fat, and resistant starch composition.

## Figures and Tables

**Table 1:** Values for pasting, cooking, and proximate composition descriptors determined for 105 pea accessions (mean; relative standard deviation, RSD; range).

Descriptor	Mean ± RSD	Range
Physicochemical parameters		
100-seed weight (g)	18 ± 28	5–29
Hydration capacity (%)	116 ± 17	62–162
Unhydrated seeds (%)	10 ± 88	2–50
Cooking time (min)	33 ± 106	5–120
Pasting viscosities (cP *)		
Peak	2643 ± 50	83–4836
Trough	2358 ± 49	58–4066
Breakdown	285 ± 67	8–867
Final	4018 ± 50	212–7471
Setback	1660 ± 54	151–3489
Basic composition (%)		
Protein	22 ± 0.1	16–30
Fibre	7 ± 0.1	6–10
Fat	2 ± 0.3	1–3
Resistant Starch	3 ± 50	1–7

* cP, centipoise.

**Table 2:** Correlation coefficients of the traits ^†^ presented in Table 1 for the 105 pea accessions analysed.

	Trough (cP)	Break (cP)	FV (cP)	SB (cP)	SW (g)	HC (%)	US (%)	CT (min)	Protein	Fibre	Fat	RS
Peak	1.000 **	0.804 **	0.989 **	0.949 **	0.218 *	−0.631 **	−0.005	−0.391 **	−0.443 **	−0.619 **	−0.759 **	−0.269 *
Trough		0.743 **	0.989 **	0.944 **	0.192 *	−0.627 **	−0.009	−0.397 **	−0.421 **	−0.613 **	−0.761 **	−0.281 *
Break			0.764 **	0.768 **	0.333 **	−0.518 **	0.015	−0.264 *	−0.475 **	−0.516 **	−0.572 **	−0.136
FV				0.982 **	0.249 **	−0.610 **	−0.031	−0.373 **	−0.495 **	−0.610 **	−0.741 **	−0.263 *
SB					0.317 **	−0.568 **	−0.059	−0.329 **	−0.577 **	−0.587 **	−0.690 **	−0.230 *
SW						−0.011	−0.214 *	−0.007	−0.408 **	−0.303 **	−0.058	−0.068
HC							−0.578 **	0.035	0.327 **	0.393 **	0.493 **	0.359 **
US								0.138	0.139	0.118	0.013	−0.055
CT									−0.130	0.362 **	0.258 *	0.146
Protein										0.211 *	0.113	0.040
Fibre											0.666 **	0.217 *
Fat												0.132

^†^ FV, final viscosity; SB, setback; SW, 100-seed weight; HC, hydration capacity; US, unhydrated seeds; CT, cooking time; RS, resistant starch; *, ** significant at the 5% and 1% levels of significance, respectively.

**Table 3:** Mean peak (cP *), trough (cP), break (cP), final (FV, cP), and setback viscosities (cP), seed weight (SW, g), hydration capacity (HC, %), unhydrated seeds (US, %), and cooking time (CT, min) traits determined for 105 pea accessions within the classes distinguished by seed shape, colour, and surface ^†^.

	Peak	Trough	Break	FV	Setback	SW	HC	US	CT
Shape									
Ellipsoid (*n* = 42)	3252 ^a^	2910 ^a^	342 ^a^	4938 ^a^	2028 ^a^	17 ^a^	109 ^a^	10 ^a^	22 ^a^
Cylindrical (*n* = 24)	3066 ^a^	2726 ^a^	340 ^a^	4632 ^a^	1906 ^ab^	19 ^a^	112 ^a^	11 ^a^	27 ^ab^
Rhomboid (*n* = 11)	1953 ^b^	1716 ^b^	236 ^ab^	2944 ^b^	1228 ^b^	16 ^a^	121 ^ab^	10 ^a^	51 ^bc^
Irregular (*n* = 16)	582 ^c^	496 ^c^	86 ^b^	918 ^c^	422 ^c^	18 ^a^	135 ^b^	13 ^a^	67 ^c^
n.d. (*n* = 12)	3046 ^a^	2759 ^a^	287 ^a^	4684 ^a^	1925 ^ab^	19 ^a^	115 ^a^	9 ^a^	16 ^ab^
Colour									
Cream yellow (*n* = 9)	3107 ^ab^	2765 ^ab^	342 ^a^	4727 ^ab^	1962 ^ab^	19 ^a^	111 ^ab^	8 ^ab^	11 ^a^
Yellow green (*n* = 24)	3120 ^a^	2777 ^a^	342 ^a^	4736 ^a^	1958 ^a^	19 ^a^	112 ^a^	9 ^a^	23 ^a^
Light green (*n* = 14)	2200 ^ab^	1933 ^ab^	267 ^a^	3365 ^ab^	1432 ^ab^	19 ^a^	126 ^ab^	8 ^a^	34 ^a^
Dark green (*n* = 8)	2677 ^ab^	2420 ^ab^	257 ^a^	3957 ^ab^	1537 ^ab^	11 ^b^	103 ^a^	15 ^ab^	51 ^a^
Green (*n* = 23)	1744 ^b^	1548 ^b^	196 ^a^	2609 ^b^	1061 ^b^	18 ^a^	128 ^b^	9 ^a^	44 ^a^
Army green (*n* = 5)	3172 ^ab^	2800 ^ab^	372 ^a^	4849 ^ab^	2049 ^ab^	16 ^ab^	99 ^a^	23 ^b^	52 ^a^
Brown (*n* = 8)	2938 ^ab^	2608 ^ab^	331 ^a^	4491 ^ab^	1883 ^ab^	17 ^ab^	103 ^a^	16 ^ab^	39 ^a^
Orange brown (*n* = 2)	3210 ^ab^	3014 ^ab^	197 ^a^	5255 ^ab^	2241 ^ab^	21 ^ab^	113 ^a^	9 ^ab^	64 ^a^
n.d. (*n* = 12)	3046 ^ab^	2759 ^ab^	287 ^a^	4684 ^ab^	1925 ^ab^	19 ^a^	115 ^ab^	9 ^ab^	16 ^a^
Surface									
Rough (*n* = 29)	1475 ^a^	1302 ^a^	172 ^a^	2249 ^a^	947 ^a^	19 ^a^	127 ^a^	11 ^a^	47 ^a^
Smooth (*n* = 64)	3097 ^b^	2761 ^b^	336 ^b^	4694 ^b^	1933 ^b^	17 ^a^	110 ^b^	10 ^a^	30 ^ab^
n.d. (*n* = 12)	3046 ^b^	2759 ^b^	287 ^ab^	4684 ^b^	1925 ^b^	19 ^a^	115 ^ab^	9 ^a^	16 ^b^

^†^ within column and seed trait, means followed by the same letter are not significantly different at *p* = 0.05; n.d., not determined; * cP, centipoise.

**Table 4:** Mean protein, fibre, fat, and resistant starch (RS) values (%) determined for pea germplasm (105 accessions) within the classes distinguished by seed shape, colour, and surface ^†^.

	Protein (%)	Fibre (%)	Fat (%)	RS (%)
Shape				
Ellipsoid (*n* = 42)	22 ± 2.0 ^a^	7 ± 0.91 ^a^	2 ± 0.41 ^a^	3 ± 1.7 ^a^
Cylindrical (*n* = 24)	22 ± 1.6 ^a^	7 ± 0.75 ^a^	2 ± 0.28 ^a^	3 ± 1.5 ^a^
Rhomboid (*n* = 11)	23 ± 3.1 ^ab^	8 ± 0.95 ^a^	2 ± 0.57 ^a^	4 ± 1.7 ^a^
Irregular (*n* = 16)	24 ± 2.1 ^b^	8 ± 0.66 ^a^	2 ± 0.42 ^a^	5 ± 1.1 ^b^
n.d. (*n* = 12)	23 ± 3.4 ^ab^	7 ± 1.3 ^a^	2 ± 0.60 ^a^	3 ± 1.7 ^a^
Colour				
Cream yellow (*n* = 9)	21 ± 1.4 ^ac^	6 ± 0.67 ^a^	2 ± 0.30 ^a^	3 ± 0.82 ^ab^
Yellow green (*n* = 24)	22 ± 1.7 ^ab^	7 ± 0.77 ^a^	2 ± 0.41 ^a^	3 ± 1.8 ^ab^
Light green (*n* = 14)	22 ± 1.7 ^abc^	8 ± 0.89 ^a^	2 ± 0.61 ^a^	5 ± 1.5 ^a^
Dark green (*n* = 8)	24 ± 2.7 ^b^	8 ± 0.75 ^a^	2 ± 0.36 ^a^	3 ± 1.0 ^ab^
Green (*n* = 23)	23 ± 1.9 ^bc^	8 ± 1. 1 ^a^	2 ± 0.53 ^a^	4 ± 1.8 ^ab^
Army green (*n* = 5)	22 ± 3.3 ^ab^	7 ± 0.71 ^a^	2 ± 0.29 ^a^	3 ± 2.2 ^ab^
Brown (*n* = 8)	22 ± 3.3 ^ab^	7 ± 0.93 ^a^	2 ± 0.35 ^a^	3 ± 1.5 ^b^
Orange-brown (*n* = 2)	21 ± 0.69 ^ab^	7 ± 0.67 ^a^	2 ± 12 ^a^	2 ± 0.40 ^ab^
n.d. (*n* = 12)	23 ± 3.4 ^ab^	7 ± 1.3 ^a^	2 ± 60 ^a^	4 ± 1.7 ^ab^
Surface				
Rough (*n* = 29)	23 ± 2.5 ^a^	8 ± 0.90 ^a^	2 ± 0.52 ^a^	4 ± 1.7 ^a^
Smooth (*n* = 64)	22 ± 1.8 ^b^	7 ± 0.93 ^a^	2 ± 0.42 ^a^	3 ± 1.6 ^a^
n.d. (*n* = 12)	23 ± 3.4 ^a^	7 ± 1.3 ^a^	2 ± 0.60 ^a^	4 ± 1.7 ^a^

^†^ within column and seed trait, means followed by the same letter are not significantly different at *p* = 0.05; n.d., not determined.

**Table 5:** The *r* and *rb* genotypes of 32 pea accessions and their starch granule phenotypes, in comparison with four control pea lines of known genotype (JI 2822, JI 1194, JI 281, and JI 399).

Accession Number	*r* Allele	*rb* Allele	Starch Granule	DNA Assayed	Surface
213	*r*	*Rb*	compound	seed meal	
222	*R*	*Rb*	simple	seed meal	
226	*R*	*Rb*	simple	leaf	
240	*R*	*Rb*	simple	seed meal	
241	*R*	*Rb*	simple	seed meal	
242	*R*	*Rb*	simple	seed meal	
244	*R*	*Rb*	simple	seed meal	
246	*R*	*Rb*	simple	seed meal	
247	*R*	*Rb*	simple	leaf	
256	*r*	*Rb*	compound	seed meal	
257	*r*	*Rb*	compound	seed meal	
258	*r*	*Rb*	compound	seed meal	
259	*r*	*Rb*	compound	seed meal	
261	*r*	*Rb*	compound	seed meal	
272	*r*	*Rb*	compound	seed meal	
273	*r*	*Rb*	compound	seed meal	
279	*r*	*Rb*	compound	seed meal	
282	*R*	*Rb*	simple	seed meal	
285	*r*	*Rb*	compound	seed meal	
286	*r*	*Rb*	compound	seed meal	
287	*R*	*Rb*	simple	seed meal	
288	*r*	*Rb*	compound	seed meal	
289	*r*	*Rb*	compound	seed meal	
291	*R*	*Rb*	simple	seed meal	
292	*r*	*Rb*	compound	seed meal	
293	*r*	*Rb*	compound	seed meal	
294	*r*	*Rb*	compound	seed meal	
295	*r*	*Rb*	compound	seed meal	
296	*r*	*Rb*	compound	seed meal	
297	*r*	*Rb*	compound	seed meal	
298	*R*	*Rb*	simple	seed meal	
299	*R*	*Rb*	simple	leaf	
Control (JI 2822)	*R*	*rb*	simple	leaf	rough
Control (JI 1194)	*r*	*Rb*	compound	leaf	rough
Control (JI 281)	*R*	*Rb*	simple	leaf	smooth
Control (JI 399)	*R*	*rb*	simple	leaf	rough

DNA was prepared from seed meal or leaf samples as indicated. The seed phenotypes of the control lines are listed.

## Supplementary Materials

The following are available online at https://www.mdpi.com/2304-8158/8/11/570/s1, Table S1: List of 105 pea accessions evaluated in 2014 in Cordoba, Spain, with the corresponding bank code number and origin.
