# Supplementary material for: Variation in Pea (Pisum sativum L.) Seed Quality Traits Defined by Physicochemical Functional Properties
Source: Foods. 2019 Nov 13;8(11):570. doi: 10.3390/foods8110570 (PMC6915640; doi:10.3390/foods8110570)
Supplement: Supplementary file 1 [file foods-08-00570-s001.pdf]

Table S1 List of 105 pea accessions evaluated in 2014 in Cordoba, Spain, with the corresponding bank code number and origin

Accession	Bank Code	Origin	Accession	Bank Code	Origin	Accession	Bank Code	Origin
**LEGPS201**	PI 109865	Venezuela	**LEGPS238**	PI 347401	India	**LEGPS275**	n.a.	Czech Republic
**LEGPS202**	PI 122442	Peru	**LEGPS239**	PI 358642	Ethiopia	**LEGPS276**	n.a.	Czech Republic
**LEGPS203**	PI 142774	Mexico	**LEGPS240**	PI 494079	Chile	**LEGPS277**	n.a.	Czech Republic
**LEGPS204**	PI 143483	Iran	**LEGPS241**	JI 502	n.a.	**LEGPS278**	n.a.	Czech Republic
**LEGPS205**	PI 143486	Iran	**LEGPS242**	JI 1213	n.a.	**LEGPS279**	n.a.	Czech Republic
**LEGPS206**	PI 162692	Argentina	**LEGPS243**	CGN16640	Sudan	**LEGPS280**	n.a.	Czech Republic
**LEGPS207**	PI 162910	Paraguay	**LEGPS244**	CGN16639	Ethiopia	**LEGPS281**	n.a.	Czech Republic
**LEGPS208**	PI 164568	India	**LEGPS245**	CGN16582	Nepal	**LEGPS282**	n.a.	Czech Republic
**LEGPS209**	PI 195405	Guatemala	**LEGPS246**	CGN03190	Turkey	**LEGPS283**	n.a.	Czech Republic
**LEGPS210**	PI 203065	Finland	**LEGPS247**	CGN03165	Turkey	**LEGPS284**	n.a.	n.a.
**LEGPS211**	PI 204667	Netherland	**LEGPS248**	CGN03229	Ethiopia	**LEGPS285**	JI 1210	France
**LEGPS212**	PI 220673	Afghanistan	**LEGPS249**	PI 311112	Guatemala	**LEGPS286**	JI 1412	n.a.
**LEGPS213**	PI 234262	USA	**LEGPS250**	PI 324705	France	**LEGPS287**	JI 1559	Mexico
**LEGPS214**	PI 254625	Finland	**LEGPS251**	PI 266070	Sweden	**LEGPS288**	JI 1747	n.a.
**LEGPS215**	PI 254626	Australia	**LEGPS252**	PI 180329	India	**LEGPS289**	JI 82	Afghanistan
**LEGPS216**	PI 262189	Costa Rica	**LEGPS253**	PI 184131	Serbia	**LEGPS290**	n.a.	n.a.
**LEGPS217**	PI 269760	UK	**LEGPS254**	JI 2480	Peru	**LEGPS291**	n.a.	n.a.
**LEGPS218**	PI 272143	Germany	**LEGPS255**	JI 1951	China	**LEGPS292**	W6 17516	USA
**LEGPS219**	PI 280621	USSR	**LEGPS256**	JI 2302	n.a.	**LEGPS293**	W6 17517	USA
**LEGPS220**	PI 312135	Guatemala	**LEGPS257**	JI 1566	n.a.	**LEGPS294**	W6 17518	USA
**LEGPS221**	PI 312136	Guatemala	**LEGPS258**	PI 608038	USA	**LEGPS295**	W6 17520	USA
**LEGPS222**	PI 314796	Australia	**LEGPS259**	PI 613100	USA	**LEGPS296**	W6 17519	USA
**LEGPS223**	PI 319373	Mexico	**LEGPS260**	n.a.	Australia	**LEGPS297**	W6 17521	USA
**LEGPS224**	PI 326194	Mexico	**LEGPS261**	n.a.	Australia	**LEGPS298**	IFPI 3365	Turkey
**LEGPS225**	PI 343962	Turkey	**LEGPS262**	n.a.	Australia	**LEGPS299**	IFPI 2370	Ethiopia
**LEGPS226**	PI 343984	Turkey	**LEGPS263**	n.a.	Australia	**LEGPS300**	PI 358608	Ethiopia
**LEGPS227**	PI 343993	Turkey	**LEGPS264**	n.a.	Australia	**LEGPS301**	PI 116056	India
**LEGPS228**	PI 347316	India	**LEGPS265**	n.a.	Australia	**LEGPS302**	PI 274584	Pakistan
**LEGPS229**	PI 347319	India	**LEGPS266**	n.a.	Australia	**LEGPS303**	n.a.	Spain
**LEGPS230**	PI 347321	India	**LEGPS267**	n.a.	Australia	**LEGPS304**	n.a.	Spain
**LEGPS231**	PI 347342	India	**LEGPS268**	n.a.	Australia	**LEGPS305**	n.a.	Spain
**LEGPS232**	PI 347348	India	**LEGPS269**	n.a.	Australia			
**LEGPS233**	PI 347366	India	**LEGPS270**	n.a.	n.a.			
**LEGPS234**	PI 347374	India	**LEGPS271**	n.a.	n.a.			
**LEGPS235**	PI 347375	India	**LEGPS272**	n.a.	Czech Republic			

**LEGPS236**	PI 347388	India	**LEGPS273**	n.a.	Czech Republic
**LEGPS237**	PI 347389	India	**LEGPS274**	n.a.	Czech Republic

n.a. – Not available
